# Dataset on optical characteristics and spectroscopic indices of dissolved organic matter of the Kara, Laptev, and East Siberian seas in August–September 2017

**DOI:** 10.1016/j.dib.2019.104562

**Published:** 2019-09-26

**Authors:** Anastasia N. Drozdova, Marat S. Puiman, Ivan N. Krylov, Svetlana V. Patsaeva, Alexander V. Shatravin

**Affiliations:** a Shirshov Institute of Oceanology, Russian Academy of Sciences, Moscow, Russia; b Department of Chemistry, Lomonosov Moscow State University, Moscow, Russia; c Department of Physics, Lomonosov Moscow State University, Moscow, Russia

**Keywords:** Dissolved organic matter, Fluorescence, Absorbance, Spectroscopic indices, Arctic

## Abstract

A total of 137 water samples to study optical properties of chromophoric dissolved organic matter were collected in the Artic shelf seas during the 69th cruise on board the R/V *Akademik Mstislav Keldysh* in August–September 2017. Sampling sites were located in the Kara, Laptev and East Siberian seas and associated to the areas influenced by terrestrial runoff of the Kolyma, Indigirka, Lena, Khatanga, Ob and Yenisei rivers. In this data article, conventional spectroscopic indices and lignin phenol concentrations, calculated on the basis of fluorescence and UV–vis absorption spectra, are presented.

**Table:** Specifications Table

Subject	Oceanography
Specific subject area	Optical properties of chromophoric dissolved organic matter
Type of data	Figure Table Text files
How data were acquired	Fluorat-02-Panorama spectrofluorometer (Lumex, Russia) and Solar PB2201 spectrophotometer (Solar, Belarus).
Data format	Raw Analyzed
Parameters for data collection	The sampling depths were chosen based on water mass changes identified with the CTD profiles. Water samples were then collected from Niskin bottles and prepared for further analysis. Fluorescence and absorption spectra were registered at room temperature in the stationary laboratory after the cruise.
Description of data collection	The absorption spectra were measured in a 3-cm or 5-cm quartz-glass cuvette using a dual-beam spectrophotometer against the pure water as a blank. The spectra were measured over the 220–700 nm spectral range in 1 nm increments. The fluorescence spectra were measured in a 1-cm quartz-glass cuvette at excitation wavelengths of 254 nm, 310 nm, and 370 nm.
Data source location	Shelf regions of the Kara, Laptev and East Siberian seas.
Data accessibility	Data on spectroscopic indices and raw spectra are provided with this article.

**Table:** 

**Value of the Data** • Spectroscopic indices represent efficient tools in tracing dissolved organic matter major sources. • Data can be used for the water mass characterization and the study of cross-frontal and vertical mixing in the mixing zones of Arctic rivers. • Under the conditions of climate change, a comparative analysis of the data is of great importance for understanding dynamics of supply and transformation of terrestrial dissolved organic matter in the Arctic basin.

## 1 Data

**Fig. 1:**
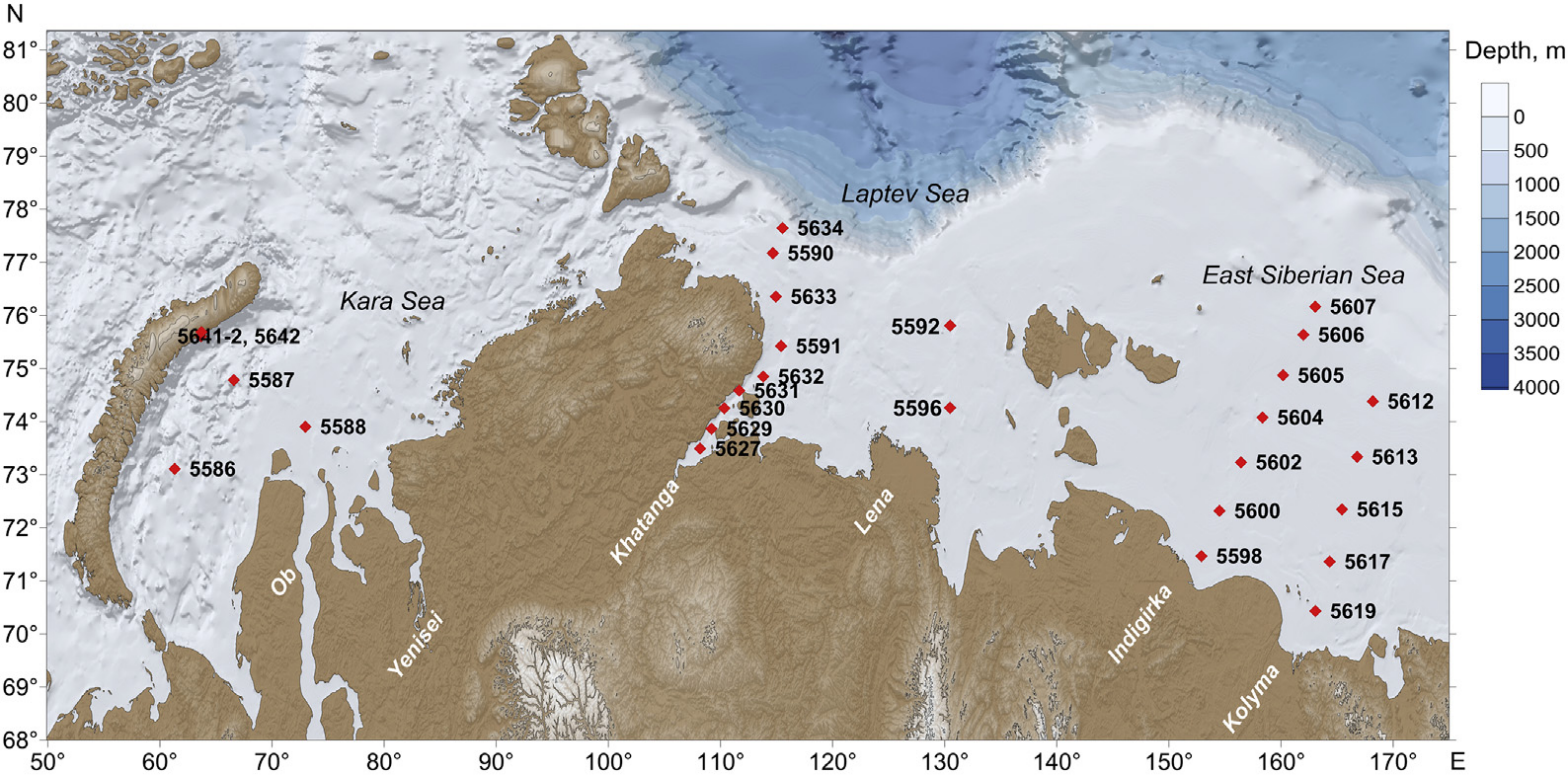
Sampling sites during the 69th cruises of the R/V *Akademik Mstislav Keldysh*.

The data presented here come from a complex study of the Arctic shelf seas conducted on board the R/V *Akademik Mstislav Keldysh* in August–September 2017 [1]. Water sampling to study the cromophoric dissolved organic mater (CDOM) optical properties was performed in the Kara, Laptev, and East Siberian seas at the sites shown in Fig. 1. The studied areas included three cross-shelf sections starting from the estuarine regions of the Khatanga, Indigirka and Kolyma rivers, as well as individual sites in the Kara and Laptev seas. Raw fluorescence and absorption spectra are given in Supplementary materials. Table 1 contains conventional spectroscopic indices, namely humification index *HIX,* index of recent autochthonous contribution *BIX*, fluorescence index *FI*, spectral slope *S*_*uvb*_, spectral slope ratio *S*_*r*_ and logarithm of lignin phenol concentration calculated on the basis of absorption spectra. The sampling depth and exact sampling date are specified.

**Table 1:** Spectroscopic indices and lignin phenol concentration of CDOM studied during the 69th cruise of the R/V *Akademik Mstislav Keldysh* in August–September 2017.

Station	mon/day/yr	Depth, m	HIX	BIX	FI [9]	FI [10]	*S*_*uvb*_, μm^−1^	*S*_*r*_	ln (TDLP_9_)
5612	09/08/2017	1	1.6	1	1.5	1.5	23.46	1.77	2.09
		10	1.84	1.01	1.27	1.69	23.67	2.07	1.94
		20	2.3	1.01	1.39	1.35	22.87	2.01	1.78
		30	1.52	0.72	1.42	1.55	21.94	2.20	2.08
		47	1.89	0.73	1.57	1.4	21.39	2.04	2.14
5613	09/08/2017	1	3.22	0.87	1.28	1.67	24.13	0.94	2.52
		8	2.85	0.87	1.14	1.52	26.11	0.98	2.32
		15	1.72	1	1.13	1.38	24.28	0.91	2.61
		29	2.68	0.95	1.65	1.74	24.22	1.12	1.97
5615	09/08/2017	1	1.49	0.95	1.47	1.24	26.21	1.39	1.97
		23	2.61	0.77	1.15	1.44	25.84	1.38	2.02
5617	09/09/2017	1	3.95	0.82	1.17	1.54	21.33	1.26	3.29
		13	1.68	0.98	1.47	1.63	25.37	1.28	2.08
		18	1.46	0.85	1.36	1.55	22.12	1.00	2.67
5619	09/09/2017	1	4.96	0.73	1.15	1.4	19.36	1.05	4.05
		14	5.16	0.82	1.29	1.35	21.81	1.36	3.04
5598	09/05/2017	1	2.85	0.8	1.14	1.55	19.57	1.08	4.09
		6	5.44	0.72	1.21	1.51	18.73	1.08	4.18
		9	6.92	0.8	1.12	1.61	20.97	1.14	3.61
		11	4.52	0.77	1.17	1.63	20.84	1.21	3.57
5600	09/05/2017	0	5	0.81	1.15	1.61	19.36	1.16	4.00
		1	5	0.81	1.15	1.61	19.48	1.21	3.97
		6	4.59	0.81	1.23	1.48	19.49	1.15	3.97
		9	5.43	0.72	1.31	1.59	21.4	1.38	3.35
		17.5	4.87	0.71	1.12	1.55	22.18	1.12	3.31
5602	09/06/2017	1	4.73	0.74	1.15	1.52	20.78	1.06	3.79
		5	n/a	n/a	n/a	n/a	20.91	1.12	3.72
		10	4.24	0.87	1.22	1.58	22.91	1.21	3.22
		16	1.66	0.81	1.36	1.73	23.3	1.24	3.15
		20	5.72	0.69	1.02	1.48	22.39	1.23	3.16
		23	3.46	0.9	1.36	1.52	21.71	1.17	3.28
5604	09/06/2017	1	2.98	0.96	1.26	1.51	22.75	1.25	3.30
		5	6.42	0.84	1.2	1.39	22.75	1.25	3.23
		11	3.36	0.9	1.41	1.7	23.4	1.24	3.13
		15	3.32	0.98	1.4	1.73	22.32	1.24	3.06
		20.5	2.87	0.93	1.14	1.52	22.68	1.31	2.94
5605	09/06/2017	0	2.93	0.97	1.23	1.33	22.8	1.04	3.00
		1	2.8	0.95	1.23	1.62	23.41	1.07	2.84
		10	3.98	0.91	1.17	1.62	22.51	1.04	2.95
		17	2.7	0.91	1.28	1.44	22.65	1.05	2.91
		33	3.64	0.9	1.17	1.69	20.97	1.07	3.16
		41	5.45	0.85	1.15	1.75	21.67	0.99	3.10
5606	09/06/2017	1	4.2	0.89	1.28	1.74	24.45	1.25	2.65
		15	4.78	0.97	1.25	1.36	23.81	1.24	2.76
		25	1.82	0.88	1.35	1.81	23.54	1.18	2.60
		33	3.69	0.86	1.22	1.63	22.84	1.18	2.67
		41	3.43	0.88	1.18	1.70	22.9	1.15	2.70
5607	09/07/2017	0	2.76	0.81	1.30	1.60	21.89	1.00	2.88
		1	4.56	0.91	1.27	1.39	21.8	1.15	3.41
		10	2.73	1.02	1.33	1.55	21.13	1.07	2.82
		20	3.32	0.92	1.14	1.51	20.39	1.12	2.80
		30	3.98	0.88	1.19	1.60	20.79	1.97	3.03
		46	3.49	0.85	1.34	1.65	19.17	1.01	1.99
		54	4.42	0.88	1.26	1.44	20.83	0.92	3.01
5586	08/27/2017	1	1.09	0.98	1.34	1.5	31.37	n/a	1.11
		25	2.21	1.1	1.4	1.65	25.01	n/a	1.42
		45	6.84	0.94	1.58	1.71	26	n/a	1.34
		55	1.68	0.94	1.3	1.55	22.79	n/a	1.34
		65	9.62	0.97	1.64	1.5	24.97	n/a	1.33
		75	2.64	0.85	1.37	2.23	24.85	n/a	1.00
		87	9.58	1.07	1.42	1.52	27.41	n/a	0.97
5587	08/28/2017	1	4.84	0.8	1.24	1.51	19.66	1.02	4.04
		5	3.97	0.8	1.17	1.46	19.48	0.98	4.08
		18	3.73	0.91	1.37	1.81	23.4	1.30	3.07
		30	2.47	0.74	1.56	1.76	26.46	1.39	1.53
		60	1.2	0.76	1.33	1.71	25.19	1.80	1.36
		150	2.08	1.09	1.44	1.95	29.32	n/a	0.85
		186	4.05	0.97	1.28	1.84	27.92	n/a	0.80
5588	08/28/2017	1	6.5	0.69	1.21	1.56	17.78	0.98	4.57
		5	6.36	0.75	1.22	1.51	17.82	0.98	4.49
		10	6.49	0.76	1.22	1.48	18.16	1.02	4.19
		20	4.49	0.9	1.2	1.76	20.99	1.17	3.38
		27	4.13	0.84	1.24	1.54	21.2	1.25	2.88
5642	09/25/2017	1	1.05	1.05	1.31	1.51	18.28	n/a	1.15
		10	1.62	0.99	1.74	1.66	17.99	n/a	1.19
		50	1.67	1.28	1.47	1.27	16.97	n/a	1.20
		110	3.11	1.03	1.52	1.47	18.63	n/a	0.85
5641_2	09/26/2017	1	1.86	0.94	1.37	1.4	22.64	1.16	2.46
		9	1.48	0.91	1.39	1.88	22.21	n/a	1.64
		15	1.78	0.97	1.52	1.52	17.74	n/a	1.44
		76	2.95	0.79	1.22	1.96	16.65	n/a	1.17
5590	08/31/2017	1	2.47	0.99	1.12	1.51	24.84	1.26	2.27
		17	2.63	0.63	1.51	1.31	25.24	1.31	2.26
		30	2.97	0.87	1.26	1.41	23.03	1.21	2.45
		40	3.16	0.82	1.35	1.54	23.64	1.23	2.25
		50	3.37	0.89	1.12	1.88	22.79	1.24	2.32
		62	2.21	0.7	1.18	1.5	22.56	1.18	2.35
5591	09/01/2017	1	5.92	0.64	1.13	1.46	17.08	0.99	4.34
		5	3.6	0.72	1.3	1.49	17.14	1.08	4.16
		10	3.3	0.81	1.11	1.66	20.25	1.39	3.17
		15	2.84	0.94	1.35	1.68	21.74	1.67	2.68
		30	3.81	0.86	1.1	1.66	21.21	1.53	2.78
		41	5.58	0.69	1.3	1.69	20.5	1.56	2.87
5627	09/17/2017	1	8.63	0.63	1.13	1.49	16.21	0.92	5.19
		5	8.83	0.64	1.14	1.35	16.03	0.94	5.21
		11	6.74	0.66	1.15	1.39	15.81	0.94	5.03
5629	09/17/2017	1	8.1	0.59	1.18	1.51	16.03	0.95	4.96
		12	6.92	0.7	1.16	1.47	15.73	0.99	4.81
		18	6.03	0.59	1.08	1.48	15.83	0.97	4.79
5630	09/17/2017	1	7.68	0.65	1.2	1.39	15.97	0.98	4.67
		14	6.04	0.7	1.14	1.5	15.98	1.00	4.59
		20	5.24	0.63	1.34	1.63	16.08	1.00	4.51
		23	5.38	0.72	1.19	1.61	16.4	1.09	4.13
5631	09/18/2017	1	7.27	0.6	1.14	1.29	16.33	0.95	4.57
		10	7.05	0.68	1.13	1.56	16.4	0.97	4.53
		25	4.71	0.75	1.14	1.43	19.33	1.14	3.22
5591_2	09/18/2017	1	3.51	0.74	1.18	1.46	17.75	1.10	4.35
		13	2.51	0.98	1.2	1.49	n/a	n/a	n/a
		41	2.13	0.84	1.37	1.5	23.25	1.14	2.66
5633	09/19/2017	1	2.94	0.78	1.01	1.39	16.67	1.32	4.21
		18	2.29	0.77	1.43	1.74	22.06	1.67	2.24
		41	2.54	0.8	1.32	1.32	23.25	1.14	2.66
5590_2	09/19/2017	1	2.54	0.84	1.4	1.59	24.42	1.20	2.59
		24	1.69	1.01	1.27	2.15	18.45	2.39	3.89
		59	1.42	0.87	1.39	1.32	24.53	1.28	2.21
5634	09/19/2017	1	1.25	0.88	1.28	1.3	16.08	n/a	3.62
		18	3.61	0.89	1.4	1.5	14.14	2.23	4.03
		80	1.53	1.01	1.18	1.90	n/a	n/a	n/a
		175	n/a	n/a	n/a	n/a	24.79	n/a	0.81
5592	09/02/2017	1	4.73	0.78	1.14	1.49	20.26	1.15	3.64
		10	5	0.81	1.24	1.46	20.35	1.14	3.67
		20	3.77	0.81	1.17	1.52	20.72	1.23	3.02
		32	3.84	0.92	1.4	1.57	23.35	1.53	2.12
		43.5	4.75	0.84	1.34	1.55	22.96	1.63	1.82
5596	09/03/2017	1	5.75	0.72	1.26	1.4	18.27	1.09	4.26
		4	6.07	0.7	1.33	1.51	18.09	1.06	4.31
		8	5.92	0.73	1.12	1.46	18.44	1.15	4.12
		17	3.81	0.84	1.33	1.83	20.7	1.32	2.75
		22	3.98	0.91	1.45	1.65	19.69	1.41	2.79
5596_2	09/14/2017	1	5.33	0.66	1.12	1.51	16.38	0.91	5.26
		5	8.05	0.71	1.17	1.42	16.43	0.98	5.08
		8	7.37	0.69	1.17	1.46	n/a	n/a	n/a
		15	3.04	0.8	1.28	1.58	19.82	1.12	3.42
		22.5	2.87	0.77	1.26	1.39	19.53	1.50	3.28
5592_2	09/14/2017	1	3.95	0.66	1.16	1.55	17.36	1.05	4.65
		20	2.69	0.85	1.25	1.57	22.5	1.34	2.97
		44	2.56	0.85	1.21	1.8	16.19	2.08	3.10

n/a – not available.

## 2 Experimental design, materials, and methods

The sampling depths were chosen based on water mass changes identified with the CTD profiles. Water samples were then collected from Niskin bottles of 5 L volume mounted on the CTD/rosette system. The samples were filtered through precombusted at 450 °C Whatman GF/F filters with a pore size of about 0.7 μm. The filtrate was collected into the acid-cleaned 30 mL glass vials and stored under dark conditions at 4 °C until further analysis.

Absorption spectra *A*_*λ*_ have been registered at room temperature of 22±2 °C with the Solar PB2201 spectrophotometer and 3 or 5 cm quartz cuvettes. The measurements were performed within the spectral range from 220 to 700 nm at 1 nm increments. The blank-corrected absorbance spectra were converted into the Napierian absorption coefficients *a*_*λ*_ according to the following equation:

(1) $$a_{\lambda }=\frac{2.303A_{\lambda }}{l},$$

where *l* - the cuvette path length in meters.

Absorption spectra within wavelngth ranges 275–295 nm and 350–400 nm and were characterized by the exponential spectral slope coefficient *S* with respect to the equation suggested by Stedmon et al. [2,3]:

(2) $$a_{\lambda }=a_{\lambda_{0}}e^{-S\left(\lambda -\lambda_{0}\right)}$$

and depicted as *S*_*uvb*_ and *S*_*uva*_, respectively. The values of *S*_*uvb*_ and *S*_*uva*_ were determined by linear regression of the log-transformed functions of absorption coefficients *a*_*λ*_ [4]. The spectral slope ratio *S*_*R*_ was calculated as follows

(3) $$S_{r}=\frac{S_{uvb}}{S_{uva}}$$

Dissolved lignin phenol concentrations TDLP_9_ were estimated according to the equations reported for the low- and high-CDOM models by Fichot and co-workers [5]:

(4) $$\text{ln(}{\text{TDLP}}_{9}\text{) }=\;{0.7672\text{a}}_{350}\;-\;0.3987\;\;\;\;\;\;\;\;\;\;\;\;\;\;\;\;\;\;\;\;{\text{a}}_{250}\text{ < }4\text{ m}-1\text{;}$$

(5) $$\text{ln(}{\text{TDLP}}_{9}\text{) }=\;-2.282\text{ln}{\text{(a}}_{350}\text{) }-\;8.209\text{ln}{\text{(a}}_{275}\text{) }+\;11.265\text{ln}{\text{(a}}_{295}\text{) }+\;2.909\;\;\;\;\;\;\;{\text{a}}_{250}\;\geq \;4\text{ m}-1.$$

Fluorescence measurements were performed with a Fluorat-02-Panorama spectrofluorometer (Lumex Instruments) equipped with a Xenon flash lamp as a light source, and a PMT as a detector of luminescence signals. A signal averaging over 20 flashes was applied in order to compensate instability of the flash lamp intensity. The accuracy of excitation and detection wavelength settings was ascertained on a basis of Xe atomic line position and estimated as ±1 nm, spectral resolution of monochromators was 5 nm. Emission scans were acquired at excitation wavelengths *λ*_*ex*_ of 254 nm, 310 nm and 370 nm. Fluorescence measurements were performed in 1 cm quartz cuvette. All the spectra were corrected for inner-filter effects. The detected fluorescence intensity at each emission wavelength was multiplied by $10^{0.5\left(D_{ex}+D_{em}\right)}$, where *D*_*ex*_ and *D*_*em*_ represent absorbances at the wavelength of excitation and emission, respectively, related to optical path of 1 cm [6].

Humification index HIX was calculated as a ratio of integral fluorescence intensities

(6) $$HIX=\frac{\sum_{435}^{480\;}I_{\lambda }}{\sum_{300}^{345}I_{\lambda }}$$

at excitation wavelength of 254 nm as suggested by Zsolnay et al. [7]. Biological/autochthonous index BIX represent a fluorescence intensity ratio

(7) $$BIX=\frac{I_{380}}{I_{430}}$$

at excitation wavelngth of 310 nm [8]. Two options for the fluorescence index were proposed by McKnight et al. [9] and Cory et al. [10]:

(8) $$FI_{\text{McKnight et al}.\;}=\frac{I_{450}}{I_{500}}\;\;,$$

(9) $$FI_{\text{Cory et al}.\;}=\frac{I_{470}}{I_{520}}.$$

In both cases the ratios of fluorescence intensities are considered for the spectra excited at 370 nm.
